# Stress during Lactation Induces Insulin Resistance Associated with an Increase in Type 1 Cannabinoid Receptors in Liver and Adipose Tissue

**DOI:** 10.1155/2019/2806519

**Published:** 2019-01-17

**Authors:** Liza Fonseca, Valeska Castillo, Carolina Aguirre, Paulo Silva, Ana M. Ronco, Miguel Llanos

**Affiliations:** ^1^Laboratorio de Nutrición y Regulación Metabólica, Instituto de Nutrición y Tecnología de los Alimentos (INTA), Universidad de Chile, Santiago, Chile; ^2^Departamento Ciencias de la Salud, Carrera de Nutrición y Dietética, Facultad de Medicina, Pontificia Universidad Católica de Chile, Campus San Joaquín, Santiago, Chile; ^3^Escuela de Nutrición, Universidad San Sebastián, Santiago, Chile

## Abstract

Several reports have shown that stress during lactation causes long-term metabolic and hormonal disruptions. In this study, we designed experiments to evaluate the effects of stress during lactation on the abundance of Type 1 cannabinoid/endocannabinoid receptors (CB1R) in epididymal fat and liver and development of insulin resistance in adult mice. During the whole lactation, male mice pups were daily subcutaneously injected (days 1–21) with a saline solution to produce a soft nociceptive stress (NS). Mice body weight and food intake were periodically evaluated. Adult animals were subsequently subjected to an insulin tolerance test and some days later sacrificed to evaluate the amount of epididymal fat and abundance of CB1R and adipophilin in liver and epididymal adipose tissue. Lipoprotein lipase (LPL) activity and circulating levels of leptin, adiponectin, and corticosterone were also evaluated. In this model, NS during lactation significantly increased the amount of epididymal fat and induced insulin resistance in adult mice. In addition, a significantly increased abundance of CB1R and adipophilin in epididymal fat and liver was observed, together with elevated circulating levels of leptin and corticosterone. Adult NS animals also had low plasmatic adiponectin and, although nonsignificant, had a sustained trend to a greater LPL activity associated with epididymal fat. These results indicate that increased abundance of CB1R in liver and epididymal fat alters tissue functionality likely associated with development of systemic metabolic alterations such as insulin resistance in adult mice. All these pathophysiological facts are long-term consequences of nociceptive stress during lactation.

## 1. Introduction

Evidence from epidemiological and animal studies indicates that critical windows, such as foetal and/or neonatal life, represent periods in which an individual's susceptibility for long-term development of health or disease could be determined or “programmed” [1]. Thus, foetal/neonatal programming is defined as the “induction, suppression, or permanent change of somatic structures by an early stimulus or insult” [2].

Stress may constitute an early stimulus leading to adiposity, overweight, and metabolic alterations in adulthood. In this regard, it has been observed that pups repeatedly subjected to a mild nociceptive stress during lactation show a significant increase in body weight and epididymal fat pads [3].

It is known that chronic stress alters physiology of different tissues through a diverse mechanism, including hyperactivation of the hypothalamus-pituitary-adrenal (HPA) axis [4]. Interestingly, the hypothalamus plays a key role integrating biochemical and behavioural components involved in energy homeostasis [5], and one of these components is the endocannabinoid system (ECS). The ECS mainly consists of Type 1 and 2 cannabinoid receptors (CB1R and CB2R), present in several tissues including the central nervous system, adipose tissue, liver, and pancreas [6–8], with their endogenous ligands, known as endocannabinoids (ECs), being arachidonoyl ethanolamide or anandamide (AEA) [9] and 2-arachidonoyl glycerol (2-AG) [10], the most studied agonists, with endocrine, autocrine, and paracrine actions [11, 12]. Finally, enzymes synthesizing and degrading ECs are also part of this system [13].

Stress elevates endocannabinoid levels in some areas of the central nervous system which in turn activate CB1R involved in the negative feedback mechanism able to repress the activity of the hypothalamus-pituitary-adrenal axis [14]. In addition, overactivation of CB1R in some peripheral tissues has been related to overweight/obesity, insulin and leptin resistance, and dyslipidaemia [15, 16].

Adipocytes express CB1R (a target for AEA); its activation is involved in adipocytes growth and differentiation, modulation of adipokines and hormones synthesis/secretion, and stimulation of lipogenesis [15, 17, 18]. In addition, a recent study [19] reported that CB1R activation in adipose tissue alters antilipolytic activity of insulin, a fact contributing to ectopic fat deposition involved in insulin resistance. Furthermore, Osei-Hyiaman et al. [20] had demonstrated that development of insulin resistance and hepatic steatosis due to a high-fat diet is associated with the presence of CB1R in liver, a conclusion obtained using a liver-specific CB1R knockout mice model. Previously, we had shown that early stress produces long-term increases in overweight, epididymal fat content, and alterations of circulating metabolic parameters in adult mice. This condition was reversed by treatment with SR141716A, the antagonist/inverse agonist of CB1R, suggesting a global involvement of these receptors in those effects [21]. In addition, treatment with AEA during lactation leads to augmented presence of CB1R in epididymal fat, a marked increase in total body fat percentage and insulin resistance in adult animals [22, 23].

With all these antecedents in mind, the aim of this study was to evaluate the effects of early postnatal nociceptive stimulation on development of insulin resistance and CB1R abundance in epididymal fat pads and liver of adult mice and its association with molecules involved in lipid storage and tissue function.

## 2. Materials and Methods

All procedures performed in this study were approved by the Bioethics' Committee for Animal Experimentation of the Institute of Nutrition and Food Technology, University of Chile, Santiago, Chile.

### 2.1. Animals

Procedures performed in this study were similar to those previously described [21]. In brief, synchronously pregnant female CD-1 mice were kept in the animal house under normal conditions of humidity and temperature (22–24°C), on a 12 : 12 h light-dark cycle. The animals had free access to purified tap water and food. A normal diet of 4 kcal/g, equivalent to 2.8 assimilated kcal/g (Champion Co, Santiago, Chile), was used during the whole study [21].

From day 16, pregnant female mice were examined daily at 9 : 00 and 19 : 00 h for the presence of pups. After 12–16 h since detection of pups, 6–8 litters of homogeneous size (12–14 pups) were put together and males were separated from females. Afterwards, six male pups showing homogeneous weights were randomly selected and assigned to a substitute mother so that pups received random cross lactation [21]. The animals were then assigned to one of the following groups:

1. Control mice: pups were removed daily for short time from home cage using a vinyl gloved hand to measure body weight.
2. Stressed, nociceptive-stimulated mice (NS mice): during the whole lactation (21 days), pups were removed daily from home cage, and each pup was gently picked up using a vinyl gloved hand, weighed, and subcutaneously injected in the back with sterile saline solution (1 *μ*l/g body weight) with a microsyringe (25-gauge needle).

Procedures were always performed by the same experimenter in a total time of 8–10 min/cage. Twice a week, the animals were transferred to clean cages, with fresh clean bedding material.

At day 21 of age, the animals were separated from their mothers, and groups of three animals were placed in new cages until adulthood. Subsequently, adult 145-day-old animals were subjected to an insulin tolerance test, and five days later, they were sacrificed according to the guidelines for rodent euthanasia provided by the American Veterinary Medical Association [24]. After sacrifice, the whole epididymal fat pads and liver were extracted from the abdominal area, weighed, and immediately frozen in liquid nitrogen for additional experiments. In addition, blood samples were obtained from the abdominal cava vein to evaluate circulating levels of some metabolic markers as further described [21, 22].

### 2.2. Body Weight and Food Intake

Body weight was recorded every 10 days. Daily food intake per cage (containing three mice) was calculated by subtracting the lost food inside the cage due to spilling in 24 hours from day 40 to 150.

### 2.3. Western Blot of CB1R and Adipophilin

For western blotting procedures, epididymal fat pads and liver from NS mice and control animals were homogenized as previously described (Heidolph homogenizer DIAX 600) in 500 *µ*l of RIPA buffer (25 mM Tris-HCl pH 7.6, 150 mM NaCl, 1% sodium deoxycholate, 0.1% SDS) in the presence of protease inhibitor cocktail (cat. no. P2714; Sigma Chem Co, USA). The homogenates containing 30 and 12 *µ*g of protein from adipose tissue and liver, respectively, were separated in a 10% SDS-polyacrylamide gel (Mini-PROTEAN III System; Bio-Rad, USA) and subsequently transferred overnight to a PVDF membrane at 4°C [22, 23]. Rabbit polyclonal antibody for CB1R (Cayman Chemical, CA, USA) and adipophilin (Thermo Fisher Scientific, USA) was used as the primary antibody, and a peroxidase-conjugated anti-rabbit antibody was used as the secondary antibody (Rockland Immunochemicals, USA); CB1R and adipophilin were visualized by chemiluminescence (Western Lightning Plus-ECL, enhanced chemiluminescence substrate; Perkin Elmer, USA). The obtained protein bands were normalized against *β*-actin expression and quantified using the Gel-Pro Analyzer 3.1 programme [22]. Appropriate positive and negative controls with brain tissue and the corresponding blocking peptide to the CB1R antibody, respectively, were also performed (Cayman Chemical, CA, USA).

### 2.4. Plasma Hormonal Levels

Corticosterone levels were determined in duplicate using a commercial ELISA kit according to the manufacturer's instruction (cat. no. 500651; Cayman Chemical, USA). Plasma leptin and adiponectin levels were assessed in duplicate using a commercial colorimetric sandwich ELISA kit (cat. no. MOB00 and MRP300, respectively; R&D Systems, USA).

### 2.5. Lipoprotein Lipase Activity of Epididymal Adipose Tissue

Epididymal fat pads, from either nonfasting animals or after 5 h fasting, were homogenized in 0.05 M sodium phosphate buffer pH 8.0, and the samples were then treated with heparin (0.5 U). After treatment, lipoprotein lipase (LPL) activity was assessed using the CONFLUOLIP™ Continuous Fluorometric Lipase Test (cat.no. PR2003; Progen Biotechnik GmbH, Germany) according to the manufacturer's protocol. Fluorescence of quenched pyrene, after adding lipase-containing samples, was measured in a kinetic assay for 2 hours, using 342 nm excitation and 400 nm emission wavelengths in a 96-well plate at 37°C. Fluorescence intensity was measured with a SpectraMax Gemini EM Microplate Reader (Molecular Devices, USA). The activity of LPL in the assay was calculated as the ratio delta fluorescence/time and was expressed as pmol/min/mg protein. Serial dilutions of the fluorescent standard were used for calibration of measurement.

### 2.6. Insulin Tolerance Test

The insulin tolerance test (ITT) was performed in 6 h fasted mice [22]. The animals were intraperitoneally injected at time 0 with recombinant human insulin (0.75 U/kg; Eli Lilly), and tail blood glucose levels were subsequently measured (basal and 15, 30, 45, and 60 min after injection). Glycemia was determined with a highly sensitive, wide-range Accu-Chek Performa glucometer (Roche Diagnostics, Mannheim, Germany). The area under the curve (AUC) between 0 and 60 min was determined using the trapezoidal method [25].

### 2.7. Statistical Analysis

Data were expressed as mean ± SEM. Shapiro–Wilk's test was first performed to evaluate normal distribution of data. The nonparametric Mann–Whitney *U* test was utilized to establish statistical differences between experimental groups. Statistical significance was set at *P* ≤ 0.05.

## 3. Results

### 3.1. Plasma Corticosterone, Leptin, and Adiponectin

First, to validate the model, plasma levels of immunoreactive corticosterone (ir-CTT) and leptin, in both NS and control mice, were measured. As expected, adult animals subjected to nociceptive stress during lactation had significantly higher levels of ir-CTT and leptin than control mice. Here, we also incorporated measurements of plasma adiponectin which is significantly lowered in NS mice (Table 1).

### 3.2. Food Intake, Body Weight, and Epididymal Fat

**Figure 1:**
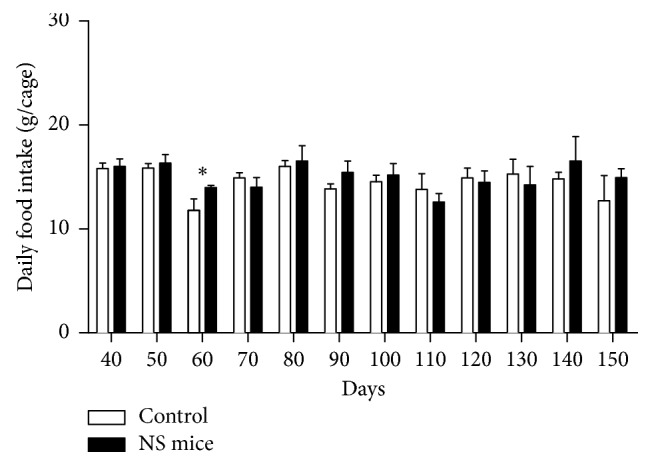
Twenty-four hours of food intake measured in gram (g), every 10 days from 40 to 150 days. ^*∗*^*P* < 0.05 (Mann–Whitney *U* test; mean ± SEM; *n*=5 cages with 3 mice each/group).

**Figure 2:**
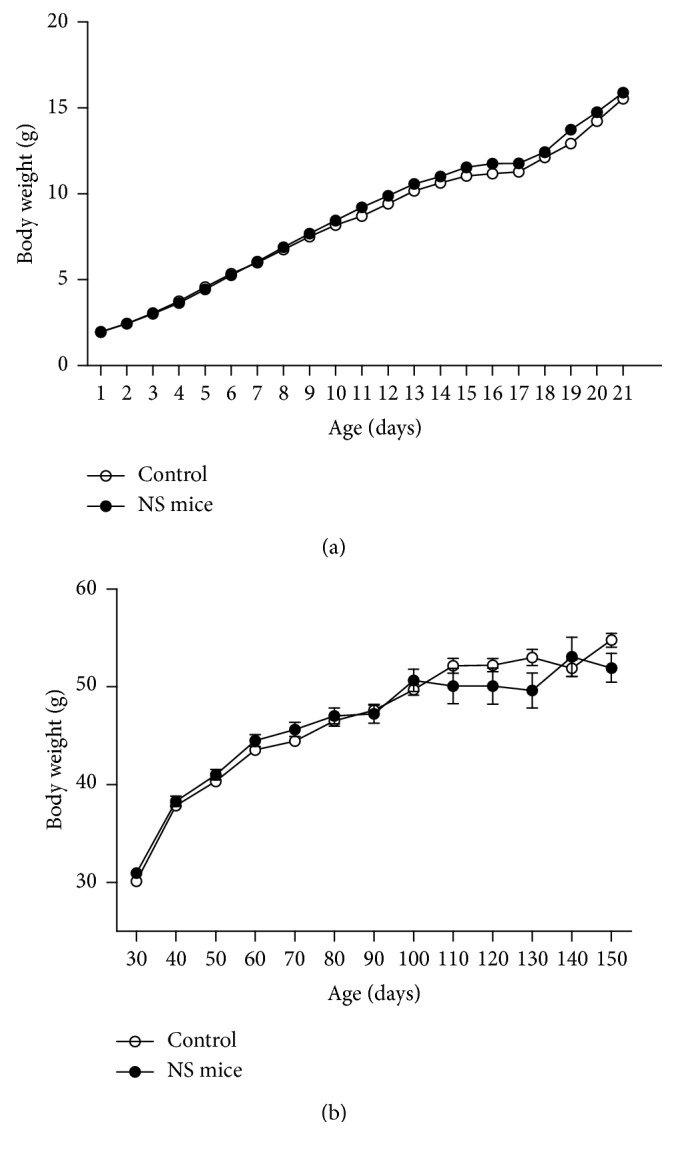
Time course of body weight of control and NS mice (a) during lactation and (b) at different ages from day 30 to 150 (mean ± SEM; *n*=14 mice/group).

**Figure 3:**
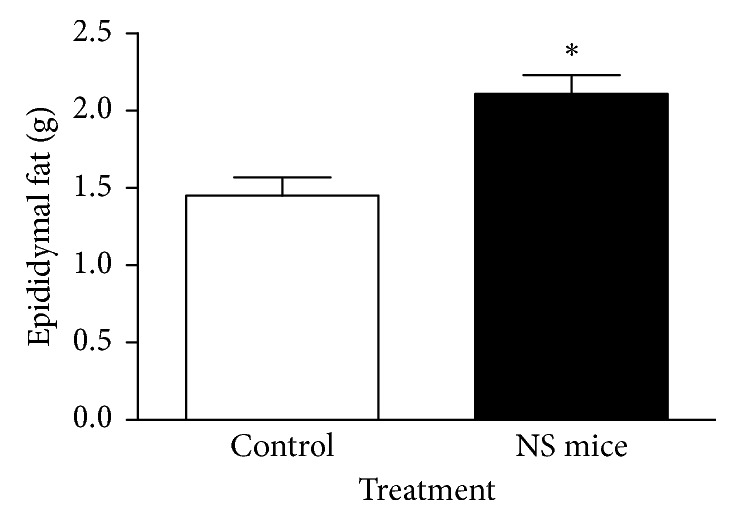
Effect of nociceptive stress during lactation on total amount of epididymal fat of adult control and NS mice. ^*∗*^*P* < 0.05 (Mann–Whitney *U* test; mean ± SEM; *n*=14 mice/group).

Daily food intake from day 40 to 150, measured in five cages (three animals per cage), show significant differences between both groups only at 60 days old (Figure 1). Body weight gain during lactation and adulthood in both groups of mice is shown in Figure 2. In this set of experiments, there were no significant differences in body weight between control and NS mice. However, in spite of no differences in body weight, 150-day-old NS mice had a marked increase in epididymal fat content (Figure 3) when compared to control mice.

### 3.3. Western Blot Analysis of CB1R and Adipophilin in Epididymal Fat Pads and Liver

**Figure 4:**
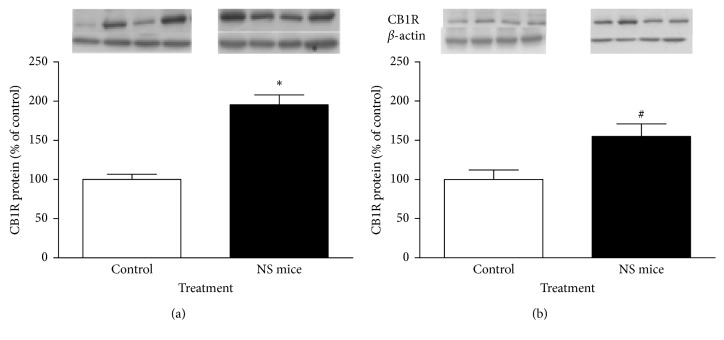
Western blot analysis of relative levels of CB1R in epididymal fat (a) and liver (b) of control and NS mice at 150 days. For densitometry quantification purposes, *β*-actin was used as a loading control. Results are expressed as percentage relative to CB1R expression in control mice. ^*∗*^*P* < 0.05; ^#^*P*=0.06 (Mann–Whitney *U* test; mean ± SEM; *n*=5 mice/group). Four representative blots are shown.

**Figure 5:**
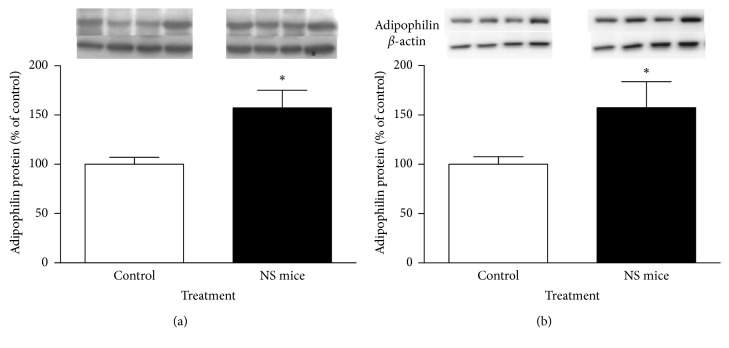
Western blot analysis of relative levels of adipophilin in epididymal fat (a) and liver (b) of control and NS mice at 150 days. For densitometry quantification purposes, *β*-actin was used as a loading control. Results are expressed as percentage relative to adipophilin expression in control mice. ^*∗*^*P* < 0.05 (Mann–Whitney *U* test; mean ± SEM; *n*=5 mice/group). Four representative blots are shown.

Figures 4(a) and 4(b) show CB1R protein levels in epididymal fat and liver of 150-day-old control and NS mice, respectively. It may be observed that NS mice almost had as much as twice the levels of CB1R (60 kDa) in epididymal fat than control animals (*P* < 0.05). In liver, there was also a 55% increase in abundance of CB1R protein, which is in the limit of significance (*P*=0.06). In addition, adipophilin (a protein marker for lipid accumulation in somatic cells; 48 kDa) was found to be about 60% higher in epididymal fat and liver of NS mice than in that of control animals (Figures 5(a) and 5(b); *P* < 0.05). In both cases, *β*-actin (42 kDa) was utilized as a loading control.

### 3.4. Lipoprotein Lipase Activity of Epididymal Fat

**Figure 6:**
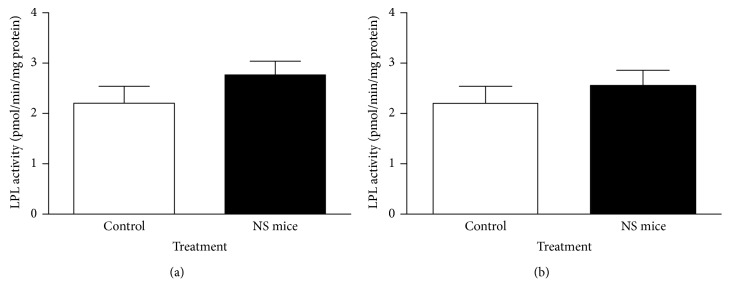
Lipoprotein lipase activity in adipocytes of epididymal fat pads from fasting (a) and nonfasting (b) adult control and NS mice. No significantly different values (Mann–Whitney *U* test; mean ± SEM; *n*=5 mice/group).

No significant differences in epididymal fat LPL activity were found between both groups of fasted animals; however, there was a consistent, although nonsignificant, higher LPL activity in NS mice, showing a sustained 25.9% increase than those control mice (Figure 6(a)). This trend was also observed in nonfasted mice although in a minor extent of 17% increase (Figure 6(b)).

### 3.5. Insulin Tolerance Test

**Figure 7:**
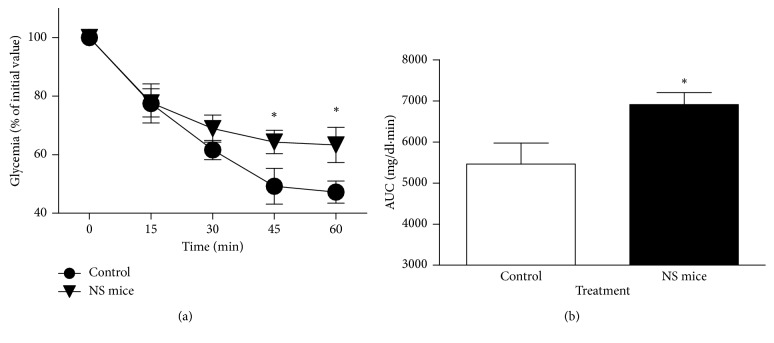
(a) Insulin tolerance test (ITT) in adult, 145-day-old control and NS mice. (b) Area under the curve of ITT obtained from both animal groups. Results are expressed as mean ± SEM (*n*=6 mice/group; ^*∗*^*P* < 0.05; Mann–Whitney *U* test).

In Figure 7(a), it is observed that adult NS mice show an insulin resistance condition which is not observed in nonstressed control mice, where glycemia reached about 50% of the initial value after 30–60 min of insulin injection, where NS mice only reached a 70%. In addition, area under the curve obtained with glycemia values from NS mice is higher and significantly different from the area obtained in control animals (Figure 7(b)).

## 4. Discussion

This study shows that stress during lactation leads to systemic insulin resistance and a significant increased abundance of CB1R in epididymal fat pads, concomitant with an increased amount of this tissue [21]. This finding, in addition to a reduced expression and activity of FAAH, previously shown in our laboratory [21, 26], indicates a concerted condition where increased levels of both, the agonist AEA (due to low FAAH) and its receptor, may likely result in overactivity of CB1R, with consequences in adipose tissue accumulation and altered physiology. In this sense, a significantly higher expression of adipophilin in epididymal fat pads and liver of stressed animals was clearly observed. This 50 kDa protein, a marker of lipid accumulation [27], is associated with the formation of adiposomes in several types of cells, having a role in the regulation of lipid storage and reserves. Recently, it has also been attributed to adiposomes, the ability to incorporate AEA, suggesting that AEA is not only released in response to demand but also complemented by its intracellular dynamic equilibrium according to transport and availability in adiposomes [11]. Thereby, the significantly higher protein levels of adipophilin found in epididymal fat and liver of stressed animals not only indicate an increased abundance of adiposomes and lipid accumulation but also indirectly suggest a higher AEA storage. In this sense, it is important to mention that a high-fat-diet-induced accumulation of lipids in adipose tissue and liver involves hyperactivation of CB1R associated with elevated levels of AEA and CB1R in both tissues [8, 15]. In addition, as already shown [21], the amount of epididymal fat becomes normal when these animals are orally treated with SR141716A, a well-known antagonist/inverse agonist of CB1R, indicating a role for these receptors in adipose tissue accumulation. Fatty acids availability for lipid synthesis and storage in adiposomes may be explained in part by increased LPL activity observed in response to CB1R stimulation [15]. In this regard, it has been shown that primary cultured adipocytes treated with a CB1R agonist (WIN-55, 212) increased LPL activity in a dose-dependent manner [15], which in turn increases availability of fatty acids to be incorporated in adipocytes. Although our present results indicate a nonsignificant 17–25% increase in LPL activity of adult NS mice, this increase is chronically sustained during nonfasting and fasting. This condition may result in a long-term higher accumulation of epididymal fat in adult NS mice, as previously observed [3, 21].

In this study, we have observed a clear state of insulin resistance in those adult mice previously stressed during lactation. In this regard, it has recently been shown that activation of CB1R affects antilipolytic activity of insulin in adipose tissue [19]. This effect may result in ectopic deposition of lipids in tissues involved in glucose homeostasis, leading to insulin resistance [28]. Interestingly, in this study, we have also observed an increase in hepatic CB1R, and previously, we showed a decreased activity of FAAH in the liver of NS mice; both conditions may converge in hyperactivation of CB1R. In this context, Osei-Hyiaman et al. [20] working in a liver-specific CB1R knockout mice model concluded that endocannabinoid activation of hepatic CB1R contributes to high-fat-diet-induced insulin resistance. Thus, NS mice insulin resistance shown in the present study may be likely associated with a chronic overactivity of CB1R in adipose tissue and liver.

In its role as an endocrine organ, adipose tissue produces the hormone adiponectin, whose plasma levels are negatively associated with obesity [29]. In this study, plasma concentrations of adiponectin in stressed animals were significantly lower than those in controls. Adiponectin has a role in reducing the expression of enzymes involved in lipogenesis and increases fatty acid oxidation in the muscle, reducing body weight in mice [30]. Interestingly, data regarding CB1R activation and decreased plasma adiponectin levels have already been reported. Thus, CB1R blocking with SR141716A in rats and adipocyte culture results in increased expression and plasma concentration of adiponectin [7].

Another hormone secreted primarily by adipose tissue is leptin, which crosses the blood-brain barrier and acts on the hypothalamus indicating body energy reserves and controlling EC levels [31, 32]. Plasma leptin levels in NS mice were significantly higher compared to those in controls, which are concordant with higher amounts of epididymal fat found, confirming the positive correlation between these two parameters.

When analyzing immunoreactive corticosterone results, plasma levels of this hormone remained high until adulthood only in NS mice. This result is in agreement with previous studies showing that postnatal daily stress induces hormonal changes and metabolic alterations that may persist during adulthood, including elevated circulating levels of corticosterone and ACTH [3]. In this sense, it has also been reported that mice chronically exposed to corticosterone develop metabolic syndrome [33]. Interestingly, it was also demonstrated that corticosterone-stimulated metabolic syndrome depends on CB1R activity. Indeed, CB1R “knockout” animals do not show such a metabolic alteration in this mouse model [33]. Moreover, we had previously shown that NS mice subsequently treated with the CB1R antagonist/reverse agonist SR141716A did not show metabolic effects of stress present in adult mice, although corticosterone levels remained high [21]. All these facts indicate a relationship between glucocorticoids and the activity of CB1R in regulating metabolism. Whether glucocorticoids influence CB1R gene and/or protein expression is a matter of present studies.

This study may have relevance to human adipose tissue depots and liver function. Indeed, significantly greater amounts of endocannabinoids have been reported in the blood and visceral fat of obese human subjects than normal subjects, likely involved in hyperactivation of CB1R, affecting hormonal and metabolic function of visceral fat, as stated above [34]. In addition, elevated circulating endocannabinoids may affect liver processes by stimulating *de novo* fatty acid synthesis and triglyceride accumulation as a first stage to develop long-term nonalcoholic fatty liver, where a role for CB1R has already been shown [35, 36]. Stressful conditions of life, via glucocorticoids elevations, may be an additional contributor to these obesity-associated pathologies by influencing levels of some ECS components, such as CB1R or enzymes involved in synthesis and/or degradation of endocannabinoids.

Finally, our study leads to suggest that increased abundance of CB1R protein, found in epididymal fat pads and liver of NS adult mice, contributes to accumulation of lipids in both tissues and long-term development of insulin resistance. However, we would like to point out that our study has some limitations due to unavailable information at present. For instance, we do not have data of anandamide levels either circulating and/or within studied tissues. This is an important issue because it is necessary to prove that anandamide is the NS-induced agonist able to activate CB1R in both, adipose tissue and liver. We have not either studies in nonstressed animals to determine whether corticosterone treatment is involved in elevated levels of anandamide or CB1R. Future studies should also include other visceral or mesenteric adipose tissue, together with some characterization of adipocytes. Most of these points undoubtedly deserve further research in this area, and part of them are under investigation in our laboratory.

## Figures and Tables

**Table 1:** Plasma levels of hormones in control and nociceptive-stimulated adult male mice (NS mice).

Plasma levels	Control	NS mice
Corticosterone (ng/ml)	23.9 ± 4.8	42.4 ± 7.3^*∗*^
Leptin (ng/ml)	4.6 ± 1.6	19.2 ± 4.0^*∗*^
Adiponectin (*μ*g/ml)	5.5 ± 0.5	4.1 ± 0.1^*∗*^

Data represent mean ± SEM of 5 mice from each group. ^*∗*^*P* < 0.05.

## Data Availability

The data used to support the findings of this study are available from the corresponding author upon request.
